# A collective tracking method for preliminary sperm analysis

**DOI:** 10.1186/s12938-019-0732-4

**Published:** 2019-11-27

**Authors:** Sung-Yang Wei, Hsuan-Hao Chao, Han-Ping Huang, Chang Francis Hsu, Sheng-Hsiang Li, Long Hsu

**Affiliations:** 1 Department of Electrophysics, National Chiao Tung University, Hsinchu, 30010 Taiwan; 2 Department of Medical Research, Mackay Memorial Hospital, Tamsui District, New Taipei City, 25160 Taiwan; 3 Mackay Junior College of Medicine, Nursing, and Management, Taipei, 11260 Taiwan

**Keywords:** Object tracking, Frame differencing, Computer-assisted sperm analyzer (CASA), Total motile sperm count (TMSC), Curvilinear velocity (VCL)

## Abstract

**Background:**

Total motile sperm count (TMSC) and curvilinear velocity (VCL) are two important parameters in preliminary semen analysis for male infertility. Traditionally, both parameters are evaluated manually by embryologists or automatically using an expensive computer-assisted sperm analysis (CASA) instrument. The latter applies a point-tracking method using an image processing technique to detect, recognize and classify each of the target objects, individually, which is complicated. However, as semen is dense, manual counting is exhausting while CASA suffers from severe overlapping and heavy computation.

**Methods:**

We proposed a simple frame-differencing method that tracks motile sperms collectively and treats their overlapping with a statistical occupation probability without heavy computation. The proposed method leads to an overall image of all of the differential footprint trajectories (DFTs) of all motile sperms and thus the overall area of the DFTs in a real-time manner. Accordingly, a theoretical DFT model was also developed to formulate the overall DFT area of a group of moving beads as a function of time as well as the total number and average speed of the beads. Then, using the least square fitting method, we obtained the optimal values of the TMSC and the average VCL that yielded the best fit for the theoretical DFT area to the measured DFT area.

**Results:**

The proposed method was used to evaluate the TMSC and the VCL of 20 semen samples. The maximum TMSC evaluated using the method is more than 980 sperms per video frame. The *Pearson* correlation coefficient (PCC) between the two series of TMSC obtained using the method and the CASA instrument is 0.946. The PCC between the two series of VCL obtained using the method and CASA is 0.771. As a consequence, the proposed method is as accurate as the CASA method in TMSC and VCL evaluations.

**Conclusion:**

In comparison with the individual point-tracking techniques, the collective DFT tracking method is relatively simple in computation without complicated image processing. Therefore, incorporating the proposed method into a cell phone equipped with a microscopic lens can facilitate the design of a simple sperm analyzer for clinical or household use without advance dilution.

## Background

Semen analysis is essential in both clinical and research settings for investigating male fertility status, which affects approximately 15% of couples throughout the USA [1]. A normal sperm concentration of semen ranges from $15 \times 10^{6}$ to $259 \times 10^{6} /{\text{ml}}$ [2]. Healthy sperms are motile in semen. Excessive immotile sperms result in male infertility. The total sperm count (TSC) and the motility, the ratio of the total motile sperm count (TMSC) to the TSC within a same volume [3], are important parameters for measuring sperm fertility.

According to the latest (2010) World Health Organization (WHO) standard for normal sperm [3], the minimal concentration and motility of total sperms have been lowered to $15.0 \times 10^{6} / {\text{ml}}$ and 40.0%, respectively [4]. The product of these two numbers, $\left( {15 \times 10^{6} / {\text{ml }} \times 40.0{\%} = 6 \times 10^{6}/ {\text{ml}}} \right),$ indicates the minimal concentration of total motile sperms for normal sperm because motility is equivalently the ratio of the concentration of total motile sperms to the concentration of total sperms of a semen [3].

Some researchers have reported that the TMSC shows a better correlation with the spontaneous ongoing pregnancy rate than the WHO 2010 classification system [5, 6]. Consequently, the TMSC [7, 8] and the curvilinear velocity (VCL) [9–11] of the motile sperms are two of the characteristic parameters of a semen sample in a preliminary semen analysis as well. Note that the VCL is the average speed of a sperm head through its real path.

Traditionally, dozens of individual sperm parameters can be evaluated using an automatic computer-assisted sperm analysis (CASA) instrument [12, 13] that tracks the trajectory of every sperm individually using a point-based tracking technique [14]. However, the individual point-tracking technique requires heavy computation for complicated image processing to detect, recognize and classify each of the target objects, individually [15]. As a semen sample is dense, severe overlapping of sperms would degrade the accuracy of CASA [16, 17]. The disadvantages make the CASA instrument expensive for clinical use and subject to semen concentration below $50 \times 10^{6} / {\text{ml}}$, as suggested by the WHO 2010 standard [3]. In other words, a semen sample of higher concentration requires advance dilution. However, not only is dilution inconvenient to the users but every dilution also increases the inaccuracy of the measurement. Recently, the overlapping problem inherited from the point-tracking technique has been greatly alleviated by using multi-target tracking algorithms [17–20]. Nevertheless, the required powerful algorithms increase the computation load.

As alternatives, inexpensive sperm analyzers are also now available [21–23]. These commercial products can analyze dense semen samples without advance dilution. However, their working mechanisms do not distinguish the motile sperms from the immotile or measure the speeds of the sperms.

In practice, many embryologists who work in the reproductive centers of hospitals or clinics still use a microscope to count the TSC manually, because manual cell counting is relatively reliable and inexpensive. Thus, manual counting is still the present gold standard method for cell counting [24–26]. However, manual counting is exhausting and time-consuming. A dense semen sample would lower the repeatability and thus the accuracy of manual counting [27].

In this paper, we proposed a collective rather than individual tracking method for estimating the total number and average speed of up to 980 motile sperms without using complicated image processing. Using a frame-differencing technique [28–30], the proposed method leads to an overall image of all of the differential footprint trajectories (DFTs) of all motile sperms and thus the overall area of the DFTs in real time. Accordingly, a theoretical DFT model was developed to formulate the overall DFT area as a function of time as well as the total number and average velocity of a group of moving objects.

In the DFT model, the problem of overlapping is treated using a statistical occupation probability. Finally, the DFT method was examined in the evaluations of TMSC and VCL using 20 semen samples and 523 synthetic bead samples. Using the least square fitting method, we obtained the optimal values of the TMSC and the average VCL that yielded the best fit for the theoretical DFT area to the measured DFT area. The results were found to be strong correlated with those obtained using manual counting and the CASA instrument used in this study, separately. Therefore, it will be shown that the proposed DFT method is simple in computation and appropriate for preliminary sperm analysis.

## Results

### Insignificant influence of Gaussian speed distribution on TMSC and VCL

From the results of the first type of simulation conducted using the first 400 synthetic bead samples, Table 1 shows the percentage errors $\left( {E_{{N\_{\text{DFT}}}} , E_{{v\_{\text{DFT}}}} } \right)$ between the optimal $\left( {N_{\text{DFT}} , v_{{{\text{ave}}\_{\text{DFT}}}} } \right)$ and the preset $\left( {N_{\text{set}} = 1000, v_{{{\text{ave}}\_{\text{set}}}} = 60 \,\upmu{\text{m/s}}} \right)$ for the four settings of $\delta_{\text{set}}$ at 0, 18, 36 and 54 µm/s under the condition of $N_{\text{set}} = 1000$, separately. Note that the simulation for each $\delta_{\text{set}}$ was conducted 100 times for the average of $\left( {N_{\text{DFT}} , v_{{{\text{ave}}\_{\text{DFT}}}} } \right)$. As the table shows, the maximal percentage errors of $E_{{N\_{\text{DFT}}}}$ and $E_{{v\_{\text{DFT}}}}$ were 7.42% at $\delta_{\text{set}} = 18$ and 2.09% at $\delta_{\text{set}} = 0$, respectively. As a whole, the overall averages of the percentage errors $\left( {E_{{N\_{\text{DFT}}}} , E_{{v\_{\text{DFT}}}} } \right)$ were (7.10% and 1.79%), respectively.

**Table 1:** Results of the first kind of simulation conducted using the first 400 synthetic bead samples: the percentage errors $E_{{N\_{\text{DFT}}}}$ and $E_{{v\_{\text{DFT}}}}$ of the total number $N_{\text{DFT}}$ and the average speed $v_{{\text{ave}}\_{\text{DFT}}}$ of a group of synthetic beads due to their *Gaussian* speed distributions set at four different standard deviations $\delta_{\text{set}}$

$\delta_{\text{set}} \,\left( {\upmu{\text{m}}/{\text{s}}} \right)$ @ $N_{\text{set }} = 1000$ $v_{\text{ave }} = 60\;\upmu{\text{m}}/{\text{s}}$	0	18	36	54	Overall average
$N_{\text{DFT}} \,\left( \# \right)$	1069.77	1074.23	1072.46	1067.35	1070.95
Standard deviation (*#*)	36.42	34.80	39.92	44.22	38.84
Percentage error $E_{{N\_{\text{DFT}}}} \,\left( {\text{\%}} \right)$	6.98	7.42	7.25	6.74	7.10
$v_{{{\text{ave}}\_{\text{DFT}}}} \left( {\upmu{\text{m}}/{\text{s}}} \right)$	61.26	60.93	60.97	61.15	61.08
Standard deviation (µm/s)	1.32	1.26	1.46	1.61	1.41
Percentage error $E_{{v\_{\text{DFT}}}} \,\left( {\text{\%}} \right)$	2.09	1.56	1.61	1.91	1.79

### DFT method as accurate as CASA in TMSC and VCL evaluations in simulation

**Fig. 1:**
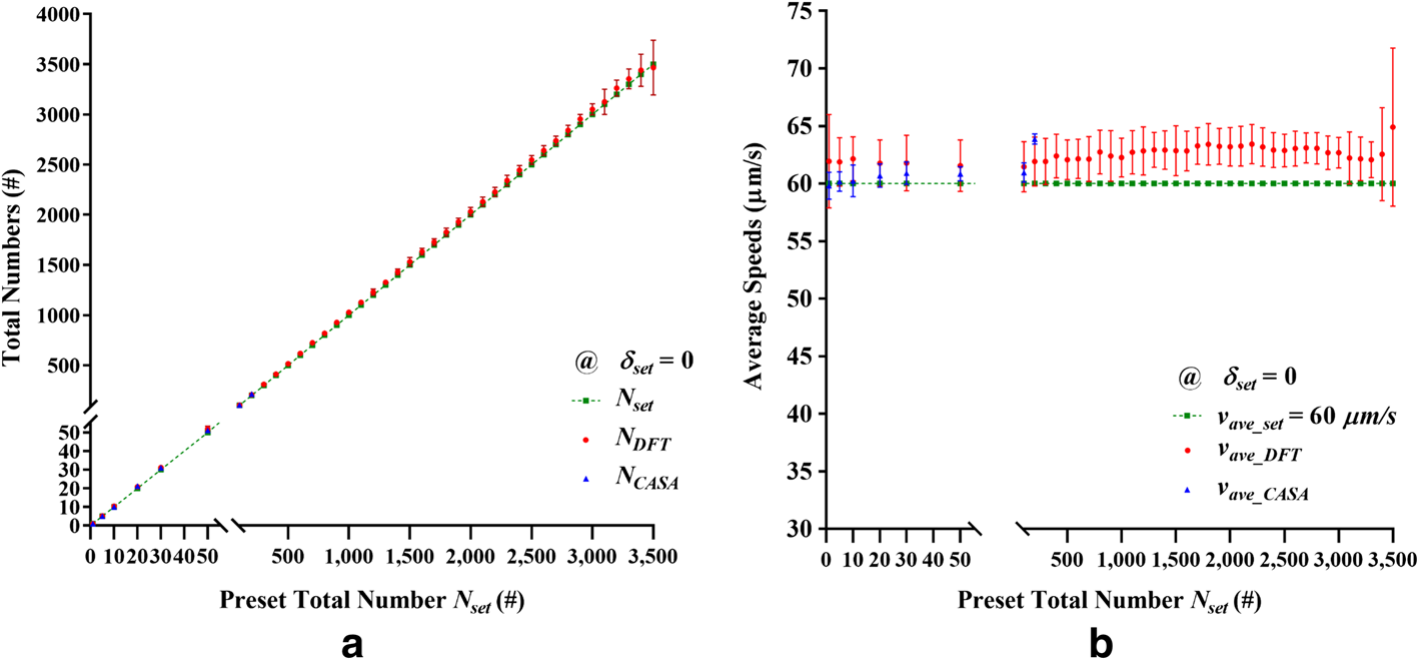
Results of the second type of simulation conducted using the 123 synthetic bead samples at 41 settings of $N_{\text{set}}$ and $\delta_{\text{set}} = 0$. **a** Total numbers $N_{\text{DFT}}$ and $N_{\text{CASA}}$ versus the preset $N_{\text{set}}$ ranging from 1 to 3500. **b** Average speeds $v_{{\text{ave}}\_{\text{DFT}}}$ and $v_{{\text{ave}}\_{\text{CASA}}}$ versus the preset $v_{\text{set}}$. Red points: $N_{\text{DFT}}$ and $v_{{\text{ave}}\_{\text{DFT}}}$ evaluated using the DFT method. Blue points: $N_{\text{CASA}}$ and $v_{{\text{ave}}\_{\text{CASA}}}$ measured using the CASA instrument. Green points: preset $N_{\text{set}}$ and $v_{\text{set}} = 60\, \upmu{\text{m/s}}$

From the results of the second type of simulation conducted using the remaining 123 synthetic bead samples, Fig. 1 shows the optimal $\left( {N_{\text{DFT}} , v_{{{\text{ave}}\_{\text{DFT}}}} } \right)$ evaluated using the DFT method, the $\left( {N_{\text{CASA}} , v_{{{\text{ave}}\_{\text{CASA}}}} } \right)$ measured by the CASA instrument, and the $\left( {N_{\text{set}} , v_{{{\text{ave}}\_{\text{set}}}} } \right)$ preset for the 41 settings of $N_{\text{set}}$ under the condition of $v_{{{\text{ave}}\_{\text{set}}}} = 60\;\upmu{\text{m/s}}$ and $\delta_{\text{set}} = 0\;\upmu{\text{m/s}}$. Specifically, Fig. 1a shows the total numbers $N_{\text{DFT}}$ and $N_{\text{CASA}}$ versus the preset $N_{\text{set}}$ that serves as the reference. It can be seen that the three series of $N_{\text{DFT}}$, $N_{\text{CASA}}$ and $N_{\text{set}}$ form a straight line. The linear correlation between any two series among $N_{\text{DFT}}$, $N_{\text{CASA}}$, and $N_{\text{set}}$ was measured by using the *Pearson* correlation coefficient (PCC) analysis [31, 32]. The resulting PCCs were found to be greater than 0.999 with the corresponding *P*‐values less than 0.001. In principle, a *P*‐value less than 0.05 is considered as statistically significant [33]. This indicates that the three series obtained using the three different methods were strongly correlated with a high statistical significance. In addition, the overall average percentage error $E_{{N\_{\text{DFT}}}}$ was 2.36% in comparison with $N_{\text{set}}$.

As for the average speed, Fig. 1b shows the average speeds $v_{{{\text{ave}}\_{\text{DFT}}}}$ and $v_{{{\text{ave}}\_{\text{CASA}}}}$ versus the preset $v_{{{\text{ave}}\_{\text{set}}}} = 60 \,\upmu{\text{m/s}}$. The overall average percentage error $E_{{v\_{\text{DFT}}}}$ was 4.35% in comparison with $v_{{{\text{ave}}\_{\text{set}}}}$.

### DFT method as accurate as CASA in TMSC and VCL evaluations in experiment

**Fig. 2:**
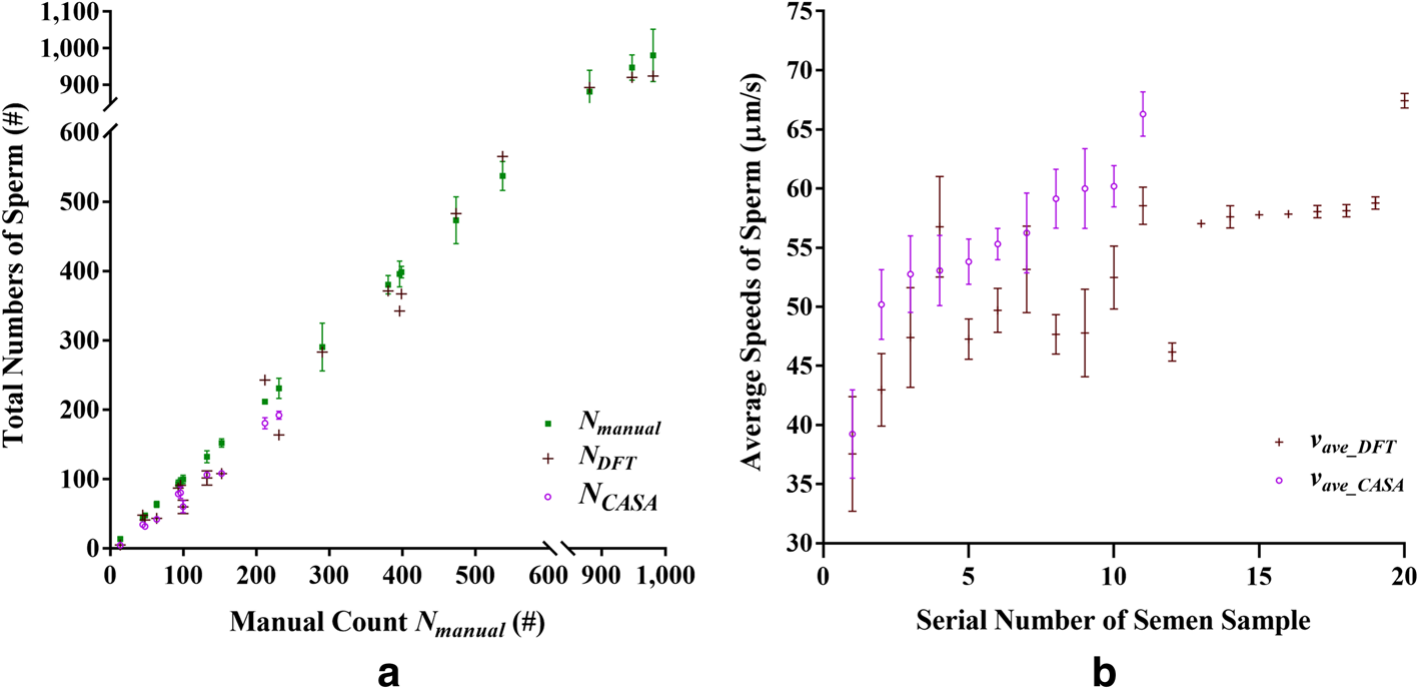
Results of the experiment conducted using 20 semen samples. **a** Total numbers $N_{\text{DFT}}$ and $N_{\text{CASA}}$ versus the manual count $N_{\text{manual}}$ that serves as the gold standard. **b** Average speeds $v_{{{\text{ave}}\_{\text{DFT}}}}$ and $v_{{\text{ave}}\_{\text{CASA}}}$ versus the serial number of the 20 semen samples. Green points: the manual counts $N_{\text{manual}}$. Dark red points: $N_{\text{DFT}}$ and $v_{{{\text{ave}}\_{\text{DFT}}}}$ evaluated using the DFT method. Violet points: $N_{\text{CASA}}$ and $v_{{\text{ave}}\_{\text{CASA}}}$ measured using the CASA instrument

From the results of the experiment conducted using the 20 human semen samples, Fig. 2 shows the optimal $\left( {N_{\text{DFT}} , v_{{{\text{ave}}\_{\text{DFT}}}} } \right)$ evaluated using the DFT method, the $\left( {N_{\text{CASA}} , v_{{{\text{ave}}\_{\text{CASA}}}} } \right)$ measured using the CASA instrument, and the $N_{\text{manual}}$ obtained by manual counting, separately. Specifically, Fig. 2a shows the total numbers $N_{\text{DFT}}$ and $N_{\text{CASA}}$ versus the manual count $N_{\text{manual}}$ that serves as the gold standard. It can be seen that the maximum $N_{\text{DFT}}$ or TMSC evaluated using the DFT method is greater than 980 sperms per video frame. In addition, the series of $N_{\text{DFT}}$ and $N_{\text{CASA}}$ are close to the series of $N_{\text{manual}}$. The PCC between the series of $N_{\text{DFT}}$ and $N_{\text{manual}}$ was found to be 0.996 with a *P*-value less than 0.001. While that between the series of $N_{\text{DFT}}$ and $N_{\text{CASA}}$ was found to be 0.946 with a *P*-value less than 0.001. As a whole, the overall average of the percentage errors $E_{{N\_{\text{DFT}}}}$ was 15.33% in comparison with the manual counts. Yet, the overall average of the coefficient of variation (CV) of the manual counting results was 5.81%.

As for the average speed, Fig. 2b shows only the average speeds $v_{{{\text{ave}}\_{\text{DFT}}}}$ and $v_{{{\text{ave}}\_{\text{CASA}}}}$ versus the serial number of the 20 semen samples, since it is difficult to estimate the speeds of sperms manually. Accordingly, the PCC between the series of $v_{{{\text{ave}}\_{\text{DFT}}}}$ and $v_{{{\text{ave}}\_{\text{CASA}}}}$ was found to be 0.771 with a *P*-value less than 0.006. It can be seen that, in general, the series of $v_{{{\text{ave}}\_{\text{CASA}}}}$ is slightly higher than that of $v_{{{\text{ave}}\_{\text{DFT}}}}$ in Fig. 2b. The series of $N_{\text{CASA}}$ is slightly lower than that of $N_{\text{DFT}}$ in Fig. 2a. This tendency will be discussed in the discussion section.

### Computation times of DFT method and CASA

**Fig. 3:**
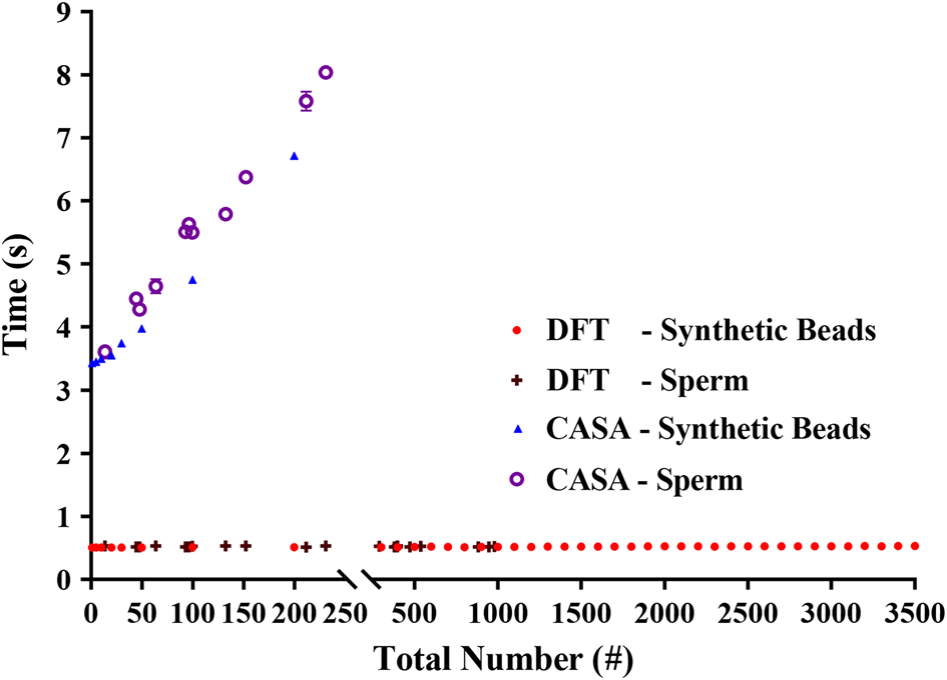
The computation times of the DFT method and the CASA instrument as a function of the total numbers of synthetic beads and sperms. Each point represents the average computation time ± standard deviations of three times of evaluations. The red points and dark red points denote the computation times of the DFT method for the synthetic beads and sperms, respectively. The blue and violet points denote the computation times of the CASA instrument for the synthetic beads and sperms, respectively

Figure 3 illustrates the computation times of the DFT method, using the collective tracking technique, and the CASA method, applying a point-tracking technique, as a function of the total numbers of synthetic beads and sperms. It can be seen that the DFT method demonstrated a larger count range of TMSC for both synthetic beads and sperms in less time than the CASA method. In addition, the computation time of the DFT method was found independent of the number of synthetic beads or sperms while that of the CASA method increased with the total numbers of synthetic beads and sperms. This supports that the collective DFT method is effective and simple in the evaluation of the total number and average velocity of a group of moving objects.

## Discussion

As shown in Table 1, the results of the first type of simulation indicated that the influence of the *Gaussian* speed distribution of a group of beads on the evaluations of $N_{\text{DFT}}$ and $v_{{{\text{ave}}\_{\text{DFT}}}}$ is insignificant in the DFT method. This can be explained in principle. Referring to Fig. 5, one can imagine that the DFT area contributed by the beads that are faster than the average speed just compensates for that contributed by the beads slower than the average speed. This suggests that the DFT method is also valid for a group of beads, such as healthy semen, with a symmetric speed distribution by assuming that they move at their average speed.

As shown in Fig. 1, the results of the second type of simulation exhibited separately that the accuracy of the DFT method in the evaluation of $N_{\text{DFT}}$ is as high as that of the CASA instrument in comparison with the preset $N_{\text{set}}$. The resulting PCCs were all greater than 0.999 with the corresponding *P*‐values all less than 0.05. The overall averages of the percentage errors $E_{{N\_{\text{DFT}}}}$ of 2.36% and $E_{{v\_{\text{DFT}}}}$ of 4.35% were all less than 5.0%. This performance persisted until there were 3500 synthetic beads for which overlapping was severe. This justifies the statistical treatment of the occupation probability for the overlapping problem in the proposed DFT model.

However, as shown in Fig. 2, the experimental results evaluated using the DFT method showed a relatively high percentage error $E_{{N\_{\text{DFT}}}}$ of 15.33% in comparison with the manual counts $N_{\text{manual}}$. The results also showed a relatively low PCC of 0.771 with a *P*‐value less than 0.05, in comparison with the average speed $v_{{{\text{ave}}\_{\text{CASA}}}}$ measured using the CASA instrument. The deterioration of the experimental results is likely due to the following three factors:

First, a finite CV of 5.81% for the series of manual counts $N_{\text{manual}}$ existed because most semen samples had high concentrations. Second, sperms slower than the cutoff speed of $5\;\upmu{\text{m/s}}$ were not counted by the CASA instrument by default. Thus, the series of $v_{{{\text{ave}}\_{\text{CASA}}}}$ tended to be slightly faster than that of $v_{{{\text{ave}}\_{\text{DFT}}}}$, as shown in Fig. 2b. The series of $N_{\text{CASA}}$ tended to be slightly less than that of *N*_DFT_, as shown in Fig. 2a. Third, the real sperms are different from the synthetic beads in size and speed distribution. The consequence is attributed to the limitation of the DFT method. As discussed above, any asymmetry on the size or speed distribution of a semen sample would degrade the accuracy of the DFT method since the TMSC and VCL of a semen sample are evaluated from the overall area of the DFT of all motile sperms in the sample. Therefore, removing the first two factors caused by the manual counting and the CASA instrument, separately, one might expect that the overall averages of the percentage errors $E_{{N\_{\text{DFT}}}}$ and $E_{{v\_{\text{DFT}}}}$ caused by the DFT method alone would be reduced.

Nevertheless, the two PCCs of 0.946 and 0.771 are all greater than 0.7, and the results of the DFT method and those of the CASA instrument are considered strong correlated [31, 32]. Consequently, the DFT method is as accurate as the CASA method in the evaluations of TMSC and VCL.

In this study, the unhealthy semen samples were found containing more slow sperms than fast ones. It can be imagined that a small or slow sperm contributes a smaller DFT area than a large one. The more the slow sperms, the smaller the overall DFT area and thus the less the TMSC in comparison with the manual count. The case of unhealthy semen elucidates the limitation of the DFT method on the symmetry of the size and speed distribution of a group of target objects. In this sense, it is recommended to carry out the simple semen analysis right after a semen sample is collected to prevent the motility of the sperms from dropping too fast [34], which will change the speed distribution of the semen sample.

## Conclusion

A collective DFT tracking method was proposed for evaluating the total number and average speed of a group of moving objects. In the DFT model, the objects are assumed to be identical in size. Their speeds can be either the same or different with a symmetric distribution. The proposed DFT method tracks the moving objects collectively and treats any overlapping using a statistical occupation probability without heavy computation. In this study, the DFT method was used to evaluate the TMSC and VCL of 523 synthetic bead samples in the simulation and 20 semen samples in the experiment. Recall that both TMSC and VCL are two characteristic parameters in preliminary sperm analysis.

The maximal count range of TMSC obtained using the DFT method is up to 980 sperms per video frame in the experiment and 3500 synthetic beads per frame in the simulation. The experimental results showed that the PCC between the two series of TMSC obtained using the DFT method and the CASA instrument was 0.946. The PCC between the two sets of VCL obtained using the method and the CASA instrument was 0.771. Their corresponding *P*-values were all less than 0.05. This indicates that the results obtained using the DFT method and those of the CASA instrument were strongly correlated with a high statistical significance [31, 32]. As a consequence, the proposed DFT method is as accurate as the CASA instrument in evaluating TMSC and VCL. This also verifies the validity of the DFT model.

In comparison with those individual point-tracking techniques, the collective DFT tracking method is relatively simple in computation without complicated image processing and provides a large count range of TMSC. Therefore, incorporating the proposed method into a microscope or a cell phone equipped with a microscopic lens can facilitate the design of a simple sperm analyzer as accurate as CASA for clinical or household use without advance dilution.

In future work, the accuracy of the TMSC and VCL can be further improved by using a 4 K $\left( {3840 \times 2160} \right)$ resolution image sensor instead of the $640 \times 480$ one since the accuracy also depends on the resolution of the video frames of a semen sample.

## Methods

The algorithm used in the proposed collective tracking method is simple. Consider a 10-s-long video film recorded with the charge-coupled device (CCD) camera inside the CASA instrument used in this study. First, two consecutive video frames of a semen sample were subtracted pixel by pixel. A unique color was marked on those resulting pixels whose absolute values are above a given threshold value. The resulting image shows the footprints of all motile sperms in a differential manner. Then, the subtraction process was repeated over a series of frames and the resulting differential footprint images were superimposed one after the other. This results in an overall image of the entire DFTs of all motile sperms.

### Color-coded DFT

**Fig. 4:**
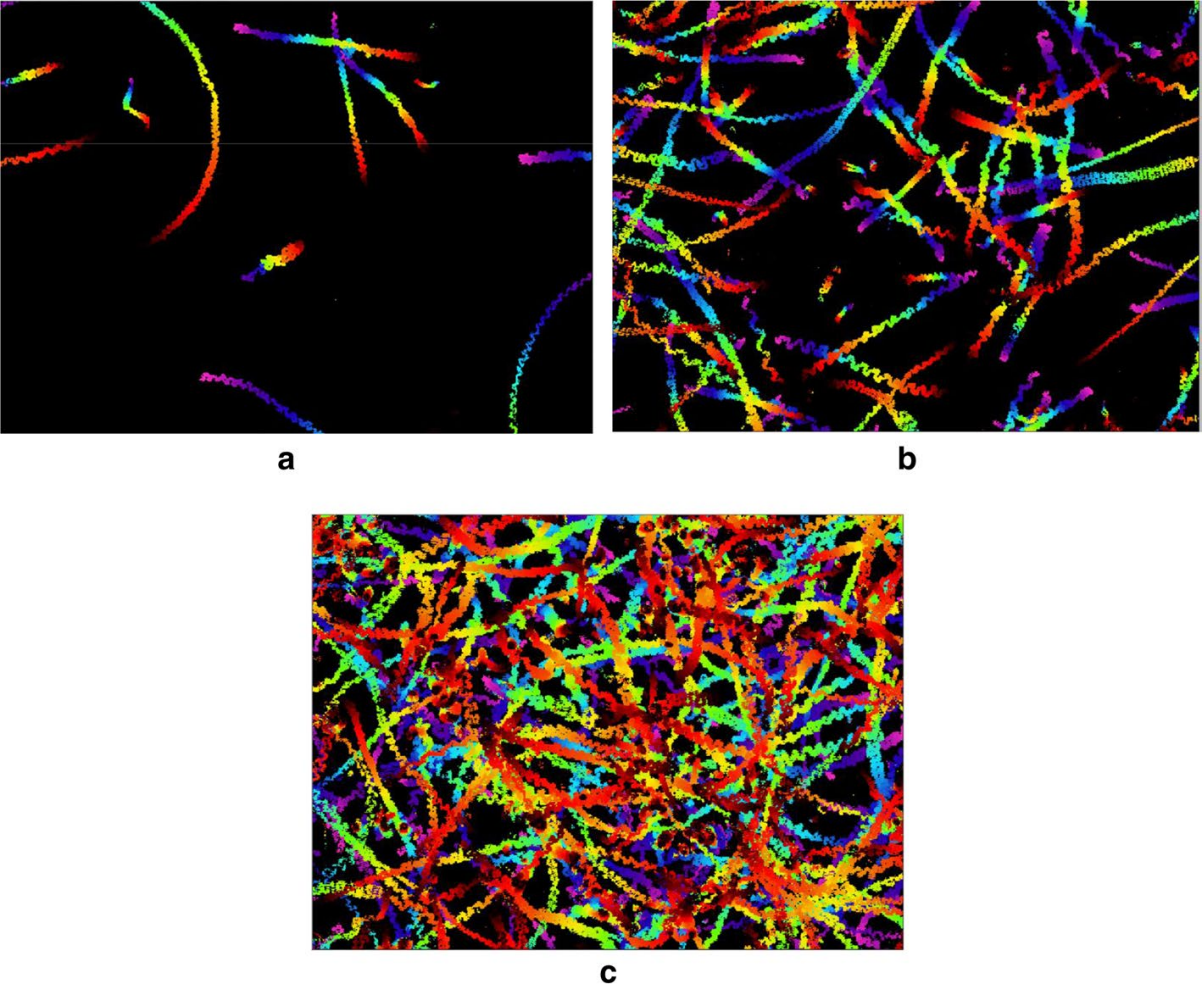
Typical color-coded DFT images of three semen samples of different concentrations for 10 s under a $10 \times$ objective lens. Each trajectory in the images represents a motile sperm. **a** TMSC = 12, **b** TMSC = 45 and **c** TMSC = 100

Figure 4 illustrates the typical color-coded DFT images of three semen samples of different concentrations for 10 s, individually. Each trajectory in the images represents a motile sperm. The longer and more colorful a trajectory is, the faster the corresponding sperm moves. As time goes by, each DFT extends and the overall area of the DFT increases with time.

In the following sections, the concept, model and procedure of the DFT method will be presented. The dependence of the overall DFT area on the total number and average speed of the moving objects is illustrated. An equation of the overall DFT area as a function of time as well as the total number and average velocity of a group of moving objects is developed. The optimal values of the total number and average speed of the moving objects can be derived using the least square fitting method. Subsequently, 20 semen samples and 523 synthetic bead samples were prepared and recorded into grayscale instead of color-coded videos for the test of the proposed DFT method in the evaluations of TMSC and VCL. The test process was conducted by comparing the results evaluated using the DFT method with those obtained using the manual count method and the CASA instrument used in the study.

### Concept: one-dimensional DFT of a single moving square

**Fig. 5:**
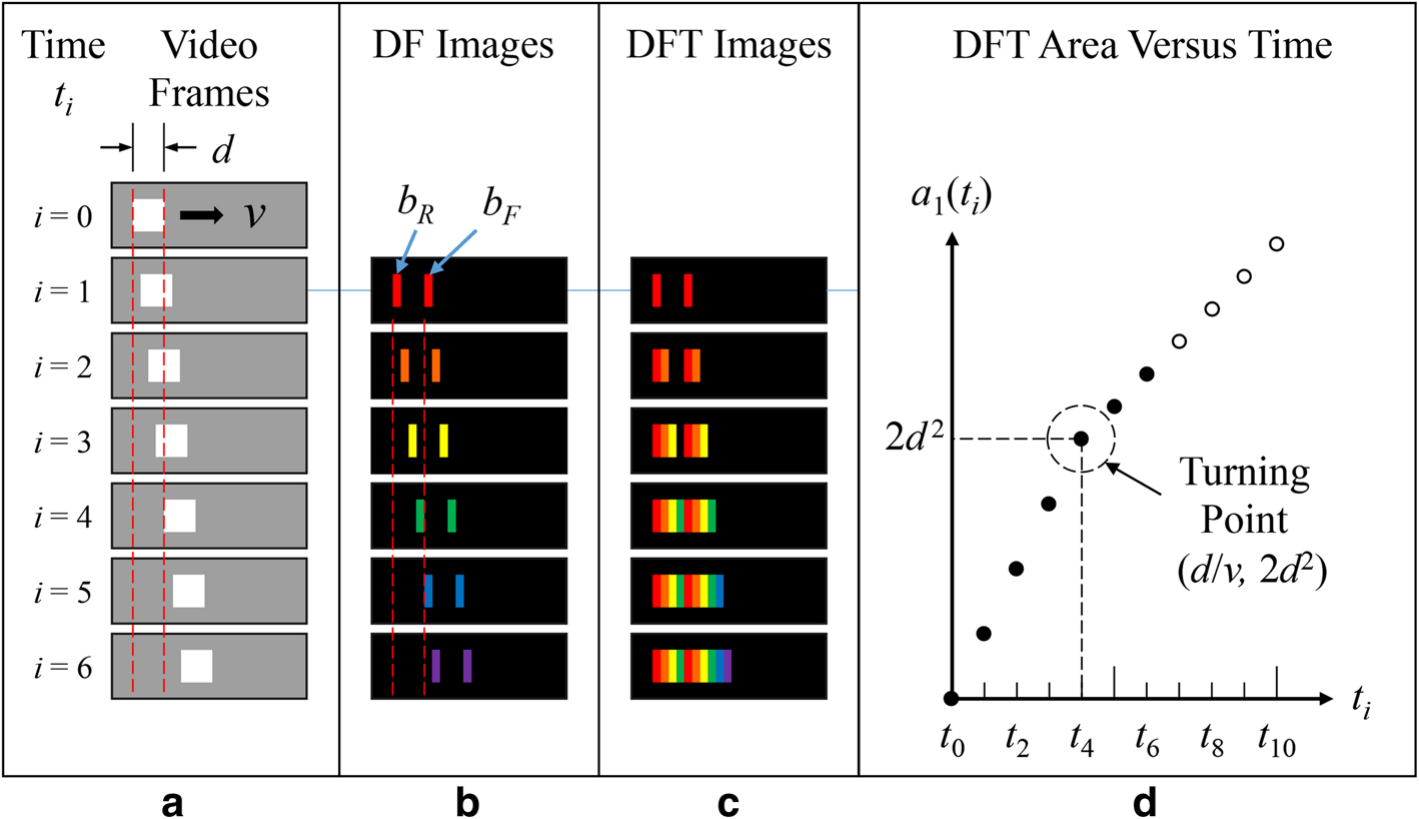
One-dimensional DFT of a single moving square. **a** The video frames of a moving square of width *d* at a constant speed *v*. **b** The differential footprint images of the square in different colors. **c** The color-coded DFT images of the square. **d** A turning point in the plot of the DFT area $a_{1} \left( {t_{i} } \right)$ as a function of time $t_{i}$

For simplicity, we first considered a square of width *d* moving at a constant speed *v* in one dimension. Figure 5a shows the continuous displacement of the square in seven consecutive video frames during a period from $t_{0} = 0$ to $t_{6} = 6\Delta t$. Herein, $t_{i} = i\Delta t.$ where $i$ is an integer representing the frame number and $\Delta t$ is a unit time interval. In this case, $d = 4v\Delta t$ is assumed such that the square moves a distance of a body length *d* at $t_{4} = 4\Delta t$. Figure 5b shows a series of six differential footprint images of the square in different colors. Each image displays a rear and a front rectangle. This is obtained by subtracting two consecutive frames and coding the resulting frame with a unique color for the pixels whose absolute values are above a given threshold value. Each pair of rectangles is called the differential footprint of the square from a single move.

Figure 5c illustrates the growth of the color-coded DFT of the moving square as a function of time by superimposing the six differential footprint images in succession. Notice that only the first four footprints appear in pairs in Fig. 5c. Subsequently, only the front rectangle of each succeeding pair appears in the DFT since the rear one always overlaps the front of a previous pair. The peculiar feature of the DFT method is reflected in Fig. 5d, a plot of the area of the DFT as a function of time. It can be seen that the DFT area $a_{1} \left( {t_{i} } \right)$ increases with time $t_{i}$ at two slopes separated at a turning point. This feature can be expressed using the following equation for $a_{1} \left( {t_{i} } \right)$:

1 $$a_{1} \left( {t_{i} } \right) = \left\{ {\begin{array}{*{20}l} {i\left( {b_{\text{R}} + b_{\text{F}} } \right)} & {{\text{for}}\;i \le \frac{d}{v\Delta t}} \\ {\frac{d}{v\Delta t}\left( {b_{\text{R}} + b_{\text{F}} } \right) + \left( {i - \frac{d}{v\Delta t}} \right)b_{\text{F}} } & {{\text{for}}\;i > \frac{d}{v\Delta t}} \\ \end{array} } \right.,$$

where the subscript “1” indicates a single square, $i$ is the video frame number, and $b_{\text{R}}$ as well as $b_{\text{F}}$ are the areas of the rear as well as the front rectangles of a differential footprint, respectively. Under the condition $b_{\text{R}} = b_{\text{F}} = dv\Delta t$, the coordinates of the turning point are $\left( {d/v,2d^{2} } \right)$. In other words, the turning point reflects the speed *v* of the moving square.

### Model: two-dimensional DFT of a group of moving beads

**Fig. 6:**
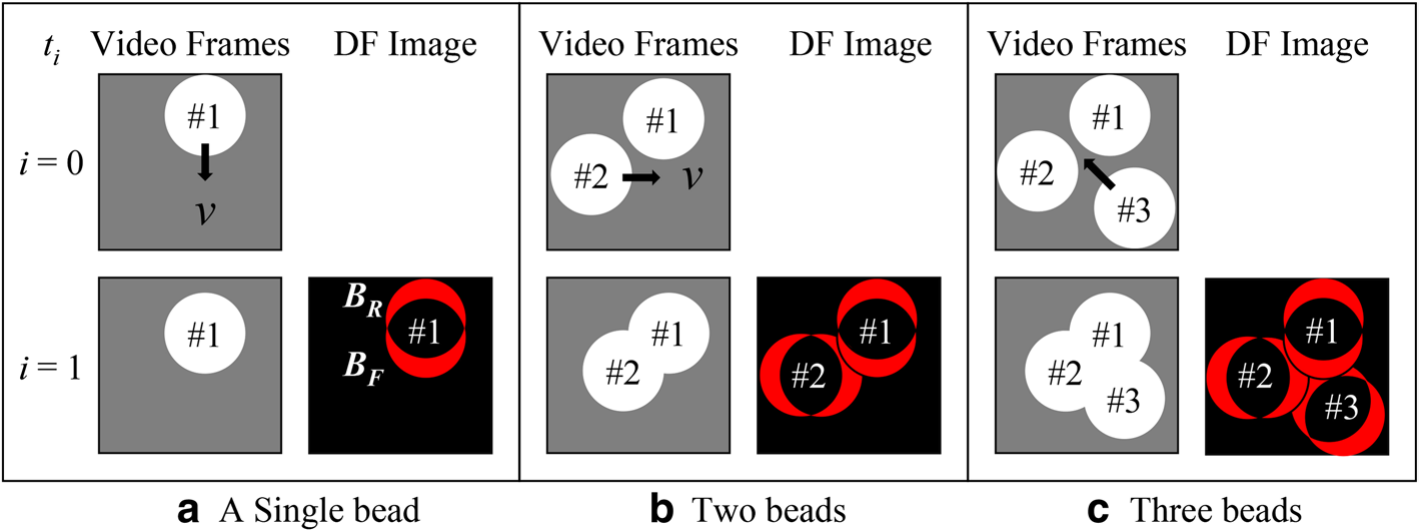
Concept of the effective footprint area of each bead in the presence of bead overlapping. **a** Effective footprint of a single bead from a move. **b** Effective footprint area occupied by two beads. **c** Effective footprint area occupied by three beads. The effective footprint areas of the respective beads are different

To be realistic, we further considered a group of identical beads with each of diameter *d* moving randomly at the same speed *v* in two dimensions. Initially, the locations and directions of movement of the beads are randomly distributed over a video frame. Figure 6 illustrates the concept of the DFT method used in developing the equation of the overall DFT area $A_{3} \left( {t_{1} } \right)$ of three beads from two consecutive video frames at $t_{0}$ and $t_{1}$ in the presence of bead overlapping. As shown in Fig. 6, each pair of crescents in red is the differential footprint of a bead. The overlapping of the three footprints changes the effective footprint area of each bead. The idea of evaluating the overall footprint area of the three beads at $t_{1}$ is to add the effective footprint area of each bead successively as follows. First, Fig. 6a shows the differential footprint of a single bead from a move. Regardless of the direction of the movement of the bead, the corresponding DFT area $A_{1} \left( {t_{1} } \right)$ of the bead is given by $A_{1} \left( {t_{1} } \right) = B_{\text{R}} + B_{\text{F}}$, where $B_{\text{R}}$ and $B_{\text{F}}$ are the areas of the rear and the front crescents, respectively. Here, the symbols $A_{1}$, $B_{\text{R}}$ and $B_{\text{F}}$ are rewritten in upper case in the model of multiple beads. Note that $B_{\text{F}} \left( { = dv\Delta t} \right)$ remains constant at all times whereas $B_{\text{R}}$ can be approximated to be the average area of the various rear crescents such that $B_{\text{R}} = \overline{{B_{\text{R}} \left( {t_{i} } \right)}} = \left( {\frac{\pi }{4}} \right)dv\Delta t.$

Second, Fig. 6b shows a statistical treatment of the overlapping of two beads. In the presence of a second bead anywhere in the frame, the probability for the second bead to occupy a full differential footprint of the area $(B_{\text{R}} + B_{\text{F}} )$ depends on the percentage of the empty area over the full area of the video frame from a statistical point of view. Accordingly, the occupation probability for a bead to occupy a full differential footprint is defined as $\left[ {1 {-} A_{\text{occupied}} /C} \right],$ where *C* is the full area of the video frame and $A_{\text{occupied}}$ is the area that has been occupied. In this case, the occupied area $A_{\text{occupied}}$ is $A_{1} \left( {t_{1} } \right)$ and $A_{1} \left( {t_{1} } \right)/C$ is the percentage of the occupied area over the frame. The resulting occupation probability is $\left[ {1 {-} A_{1} \left( {t_{1} } \right)/C} \right]$. Thus, the effective area occupied by the differential footprint of the second bead becomes $\left( {B_{\text{R}} + B_{\text{F}} } \right)\left[ {1 - A_{1} \left( {t_{1} } \right)/C} \right]$, which is the product of the full area $(B_{\text{R}} + B_{\text{F}} )$ and the occupation probability. Statistically, the overall DFT area $A_{2} \left( {t_{1} } \right)$ of the two beads is the sum of the two effective areas of the first and the second beads, as given by:

2 $$\begin{aligned} A_{2} \left( {t_{1} } \right) & = A_{1} \left( {t_{1} } \right) + \left( {B_{\text{R}} + B_{\text{F}} } \right)\left[ {1 - \frac{{ A_{1} \left( {t_{1} } \right)}}{C}} \right] \\ & = \left( {B_{\text{R}} + B_{\text{F}} } \right)\left\{ {1 + \left[ {1 - \frac{{\left( {B_{\text{R}} + B_{\text{F}} } \right)}}{C}} \right]} \right\} \\ \end{aligned} .$$

Third, Fig. 6c shows a simple geometric progression formula for the overlapping of three beads. Similarly to the statistical treatment of two beads, the presence of the third bead creates an additional effective area $\left( {B_{\text{R}} + B_{\text{F}} } \right)\left[ {1 - A_{2} \left( {t_{1} } \right)/C} \right]$. Thus, the overall DFT area $A_{3} \left( {t_{1} } \right)$ of the three beads can be expressed as:

3 $$\begin{aligned} A_{3} \left( {t_{1} } \right) & = A_{2} \left( {t_{1} } \right) + \left( {B_{\text{R}} + B_{\text{F}} } \right)\left[ {1 {-}\frac{{ A_{2} \left( {t_{1} } \right)}}{C} } \right] \\ & = \left( {B_{\text{R}} + B_{\text{F}} } \right)\left\{ {1 + \left[ {1 - \frac{{\left( {B_{R} + B_{F} } \right)}}{C}} \right] + \left[ {1 - \frac{{\left( {B_{\text{R}} + B_{\text{F}} } \right)}}{C}} \right]^{2} } \right\}. \\ \end{aligned}$$

It is worth noting that $A_{3} \left( {t_{1} } \right)$ is in the form of a geometric series and thus can be expressed in compact form as $A_{3} \left( {t_{1} } \right) = C\left\{ {1 - \left[ {1 - \left( {B_{\text{R}} + B_{\text{F}} } \right)/C} \right]^{3} } \right\}$. Further, the overall DFT area $A_{N} \left( {t_{1} } \right)$ of *N* beads at $t_{1}$ can be expressed as

4 $$A_{N} \left( {t_{1} } \right) = C\left\{ {1 - \left[ {1 - \frac{{B_{\text{R}} + B_{\text{F}} }}{C}} \right]^{N} } \right\}.$$

Based on the same idea, for the succeeding video frame at time $t_{2}$, the overall DFT area $A_{N} \left( {t_{2} } \right)$ of the same number *N* of beads can be expressed in the form:

5 $$A_{N} \left( {t_{2} } \right) = C\left\{ {1 - \left[ {1 - \frac{{B_{\text{R}} + B_{\text{F}} }}{C}} \right]^{2N} } \right\} .$$

Similarly to the turning point for a single moving square, as shown in Eq. (1) for $a_{1} \left( {t_{i} } \right)$, $A_{N} \left( {t_{i} } \right)$ also presents a turning point for *N* moving beads. It can be imagined that both crescent areas $B_{\text{R}}$ and $B_{\text{F}}$ of each bead contribute to the overall DFT area before the bead moves a distance of a body length *d* for $t_{i} \le d/v$ or $i \le d/\left( {v\Delta t} \right)$. Subsequently, for $t_{i} > d/v$ or $i > d/\left( {v\Delta t} \right)$, only the front crescent area $B_{\text{F}}$ of each bead contributes to the overall DFT area. As a consequence, it can be shown that the overall DFT area $A_{N} \left( {t_{i} } \right)$ of *N* identical beads in a video frame at time $t_{i}$ can be expressed as Eq. (6).

6 $$A_{N} \left( {t_{i} } \right) = \left\{ {\begin{array}{*{20}l} {C\left\{ {1 - \left[ {1 - \frac{{B_{\text{R}} + B_{\text{F}} }}{C}} \right]^{iN} } \right\}} & {{\text{for}}\; i \le \frac{d}{v\Delta t}} \\ {C\left\{ {1 - \left[ {1 - \frac{{B_{\text{R}} + B_{\text{F}} }}{C}} \right]^{{\frac{dN}{v\Delta t}}} } \right\} + C\left[ {1 - \frac{{B_{\text{R}} + B_{\text{F}} }}{C}} \right]^{{\frac{dN}{v\Delta t}}} \left\{ {1 - \left[ {1 - \frac{{B_{\text{F}} }}{C}} \right]^{{\left( {i - \frac{d}{v\Delta t}} \right)N}} } \right\}} & {{\text{for}}\;i > \frac{d}{v\Delta t}} \\ \end{array} } \right..$$

Indeed, the overall DFT area $A_{N} \left( {t_{i} } \right)$ is a function of time $t_{i}$, the total number *N* and the average velocity *v*. Here, *N* and *v* serve as implicit parameters. Under the condition $\left( {B_{\text{R}} + B_{\text{F}} } \right)/C \ll 1$ for small beads, Eq. (6) for $A_{N} \left( {t_{i} } \right)$ can be simplified in the following form, by making use of *Taylor*’*s* expansion technique,

7 $$A_{N} \left( {t_{i} } \right) = \left\{ {\begin{array}{*{20}l} {iN\left( {B_{\text{R}} + B_{\text{F}} } \right)} & {{\text{for}}\;i \le \frac{d}{v\Delta t}} \\ {\frac{dN}{v\Delta t}\left( {B_{\text{R}} + B_{\text{F}} } \right) + \left( {i - \frac{d}{v\Delta t}} \right)NB_{\text{F}} } & {{\text{for}}\;i > \frac{d}{v\Delta t}} \\ \end{array} } \right..$$

Notice that Eq. (6) resembles Eq. (1) as $A_{N} (t_{i} ) \approx Na_{1} (t_{i} )$ when the square in Eq. (1) is replaced with a bead. The consequence of a linear relation between multiple beads and a single bead is reasonable since bead overlapping is negligible in a group of small beads that are sparsely distributed. The applicability of Eq. (7) to small beads supports the statistical treatment of occupation probability for the bead overlapping problem in deriving Eq. (6) for $A_{N} (t_{i} ).$

### Effect of Gaussian speed distribution on TMSC and VCL

In reality, the motile sperms in a semen sample [35] move at various speeds that follow a *Gaussian*-like symmetric probability distribution [36]. It is to be noted that the probability density of a *Gaussian* speed distribution is given by:

8 $$f\left( v \right) = \frac{1}{{\delta \sqrt {2\pi } }}e^{{ - \frac{{\left( {v - v_{\text{ave}} } \right)^{2} }}{{2\delta^{2} }}}} ,$$

where $v_{\text{ave}}$ is the average speed and *δ* is the standard deviation of the *Gaussian* speed distribution. Thus, it is too complicated to develop further an analytic equation for the DFT area as a function of time in this case. Instead, through simulation, the overall DFT area $A_{N} \left( {t_{i} } \right)$ versus time $t_{i}$ was plotted for a group of *N* beads whose speeds follow a *Gaussian* distribution, as discussed above. For comparison, a graph of $A_{N} \left( {t_{i} } \right)$ versus $t_{i}$ was also plotted for the same *N* beads but with all beads moving at the same average speed $v_{\text{ave}}$ of the *Gaussian* speed distribution. The simulation results demonstrated later will show that the two plots are nearly the same. Consequently, this allows us to use Eq. (6) for $A_{N} \left( {t_{i} } \right)$ in the analysis of real semen samples.

### Procedure of DFT method

Similarly to the procedure for a single moving square as illustrated in Fig. 5, the procedure of the DFT method for a target semen sample or synthetic bead sample was as follows. First, a series of 30 consecutive grayscale video frames of the target sample were acquired, as shown in Fig. 5a. In this study, the resolution of the video frames was $640 \times 480$ pixels. The gray levels of the video frames ranged from 0 to 255. Second, any two consecutive frames were subtracted pixel by pixel. A new number “255” was assigned on those resulting pixels whose absolute values were above a threshold value, 20 in this case, and a new number “0” was assigned elsewhere. This marked a differential footprint image of all motile sperms or synthetic beads, as shown in Fig. 5b. Third, the subtraction process was repeated through the 30 consecutive frames in sequence and the resulting differential footprint images were superimposed in succession. This resulted in 29 images of all of the DFTs of all sperms or beads at different times, showing the extension of the entire DFTs as a function of time, as shown in Fig. 5c. The total number of the pixels whose values were not “0” on a DFT image at time $t_{i}$ was just the overall area $A_{\text{DFT}} \left( {t_{i} } \right)$, in pixels, of all of the DFTs on that image. Fourth, the overall DFT area of each of the 29 DFT images, $A_{\text{DFT}} \left( {t_{i} } \right)$ for $1 \le i \le 29$, was calculated and plotted versus time, as shown in Fig. 5d. Last, the least square fitting method was used to obtain the optimal values of $N_{\text{DFT}}$ and $v_{{\text{ave}}\_{\text{DFT}}}$ that yielded the best fit for Eq. (6) for the theoretical $A_{\text{DFT}} \left( {t_{i} } \right)$ to the above measured $A_{\text{DFT}} \left( {t_{i} } \right)$.

The above image process is collective rather than individual. It does not need to recognize and classify each of the target objects, individually. Thus, the DFT method is simple in computation without complicated image processing.

### CASA settings

The CASA instrument used in the study (IVOS CASA System manufactured by *Hamilton*-*Thorne* Research) was set as follows: negative phase-contrast optics and recording at the frame rate: 60 Hz; frames acquired: 30; magnification: 10× objective; minimum contrast: 80; minimum cell size: 3 µm; non-motile head size: 5 µm; non-motile head intensity: 160; medium average path VCL cutoff: 25 µm/s; low path VCL cutoff: 5 µm/s; and threshold straightness: greater than 80% [37].

### Manual counting

Each semen sample was counted three times for averaging by three researchers trained at Mackay Memorial Hospital Reproductive Medicine Center, New Taipei City, Taiwan. The use of semen for research had been reviewed and approved by the hospital’s institutional review board with the approval number 14MMHIS280.

### Experiments and simulations

Twenty semen samples and 523 synthetic bead samples were used to verify the validity of the proposed DFT method in the evaluations of TMSC and VCL. In particular, two types of simulations were conducted using the 523 synthetic bead samples for two purposes. One was to estimate the influence of the speed distribution of a group of synthetic beads on the evaluation of the total number of beads in the proposed DFT method. The other was to evaluate the influence of the total number of beads on the accuracies of the DFT method on the evaluations of the total number and average speed of the beads.

In the first type of simulation, $\delta_{\text{set}}$ was set to 0, 18, 36 and 54 at the preset $N_{\text{set}} = 1000$ and $v_{{\text{ave}}\_{\text{set}}} = 60 \;\upmu{\text{m/s}}$, separately. For each setting of $\delta_{\text{set}}$, the optimal values of $N_{\text{DFT}}$ and $v_{{\text{ave}}\_{\text{DFT}}}$ of the corresponding synthetic bead sample in a video were first evaluated following the procedure of the DFT method. Then, the optimal $\left( {N_{\text{DFT}} , v_{{\text{ave}}\_{\text{DFT}}} } \right)$ was compared with the preset $(N_{\text{set}} = 1000,$ $v_{{\text{ave}}\_{\text{set}}} = 60\; \upmu{\text{m/s}})$ and the percentage errors $E_{{N\_{\text{DFT}}}} \equiv \left( {N_{\text{DFT}} - N_{\text{set}} } \right)/ N_{\text{set}}$ and $E_{{v\_{\text{DFT}}}} \equiv \left( {v_{{{\text{ave}}\_{\text{DFT}}}} - 60} \right)/60$ were obtained, separately. Recall that $\delta_{\text{set}} = 0$ corresponds to all beads moving at a single speed $v_{\text{ave}}$ in the DFT model. As a consequence, the percentage errors $\left( {E_{{N\_{\text{DFT}}}} , E_{{v\_{\text{DFT}}}} } \right)$ at various $\delta_{\text{set}}$ reflect the influence of the speed distribution of the beads on the evaluations of $N_{\text{DFT}}$ and $v_{{\text{ave}}\_{\text{DFT}}}$ in the DFT method.

In the second type of simulation, $N_{\text{set}}$ was set to 41 different values ranging from 1 to 3500 at $\delta_{\text{set}} = 0$ and $v_{{{\text{ave}}\_{\text{set}}}} = 60\;\upmu{\text{m/s}}$, separately. For each setting of $N_{\text{set}}$, the optimal $N_{\text{DFT}}$ and $v_{{\text{ave}}\_{\text{DFT}}}$ of the synthetic bead sample in a video were first evaluated following the procedure of the DFT method. Then, these synthetic videos were sent to the CASA instrument to measure the total number $N_{\text{CASA}}$ and the VCL $v_{{\text{ave}}\_{\text{CASA}}}$ of the $N_{\text{set}}$ beads for comparison. Thus, the corresponding percentage errors $E_{{N\_{\text{CASA}}}} \equiv \left( {N_{\text{CASA}} - N_{\text{set}} } \right)/N_{\text{set}}$ and $E_{{v\_{\text{CASA}}}} \equiv \left( {v_{{\text{ave}}\_{\text{CASA}}} - 60} \right)/60$ were obtained. As a consequence, the errors $\left( {E_{{N\_{\text{DFT}}}} , E_{{v\_{\text{DFT}}}} } \right)$ and $\left( {E_{{N\_{\text{CASA}}}} , E_{{v\_{\text{CASA}}}} } \right)$ reflect the accuracies of the DFT method and the CASA instrument on the evaluations of $\left( {N_{\text{DFT}} , v_{{\text{ave}}\_{\text{DFT}}} } \right)$ and $\left( {N_{\text{CASA}} , v_{{\text{ave}}\_{\text{CASA}}} } \right)$, respectively.

To reduce uncertainties, every simulation of the first type was conducted 100 times for the average of $\left( {N_{\text{DFT}} , v_{{\text{ave}}\_{\text{DFT}}} } \right)$. Thus, 400 different bead samples were synthesized and analyzed for the four settings of $\delta_{\text{set}}$ at $N_{\text{set}} = 1000$ and $v_{{{\text{ave}}\_{\text{set}}}} = 60\;\upmu{\text{m/s}}$. In addition, every simulation of the second type was conducted three times for the averages of $\left( {N_{\text{DFT}} , v_{{\text{ave}}\_{\text{DFT}}} } \right)$ and $\left( {N_{\text{CASA}} , v_{{\text{ave}}\_{\text{CASA}}} } \right)$. Thus, 123 different bead samples were synthesized and analyzed for the 41 settings of $N_{\text{set}}$ at $\delta_{\text{set}} = 0$ and $v_{{{\text{ave}}\_{\text{set}}}} = 60\;\upmu{\text{m/s}}$. In total, 523 synthetic bead samples were analyzed in the two types of simulations. On the other hand, every semen experiment was conducted three times for the averages of $\left( {N_{\text{DFT}} , v_{{\text{ave}}\_{\text{DFT}}} } \right)$, $\left( {N_{\text{CASA}} , v_{{{\text{ave}}\_{\text{CASA}}}} } \right)$, and $N_{\text{manual}}$. Thus, the 20 different semen samples were analyzed 60 times in total.

### Sperm preparation

Twenty human semen samples were selected from 31 subjects for a wide distribution of TMSCs and VCLs of the sperms from unhealthy to healthy. The semen samples were collected by masturbation after 2 to 3 days of sexual abstinence. After liquefaction for at least 30 min, routine semen analysis was conducted according to the WHO 2010 criteria [3]. The semen samples were assessed by loading 28 µl of each sample into a 20 µm deep *Leja* slide with no dilution in any buffer. Then, the slides were inserted into the CASA instrument for semen analysis and recording, individually. After the experiment, all sperm samples were destroyed by using high-temperature steam sterilization. The process of each semen sample from collection to sterilization lasted within an hour.

### Videos of semen samples

The movements of the motile sperms within each slide in the CASA were simultaneously recorded into a grayscale video for 2 s at a frame rate of 60 Hz by using the charge-coupled device camera (CCD; Sony XC-75 CE N50) inside the CASA instrument. The pixel size of the CCD camera is 8.6 µm (horizontal) × 8.3 µm (vertical). The resolution of the video frame was $640 \times 480$ pixels. The magnification of the objective in the CASA was $10 \times$. The field of view of the CCD was about $550 \times 398$ µm^2^. Thus, under the magnification of $10 \times$, the image of a normal sperm of $4.3 \pm 0.6$ µm in length [38] was nearly 43.0 µm long upon the image sensor of the CCD and occupied an area of $5 \times 5$ pixels, approximately. Afterward, the videos of the 20 semen samples were used to verify the validity of the proposed DFT method.

### Simulation settings

Five hundred and twenty-three synthetic bead samples were arranged to cover a wide distribution of total numbers and speeds of the synthetic beads as follows. The diameters of the synthetic beads were fixed at 4.3 µm, as long as that of a normal sperm [38]. Correspondingly, the synthetic image sizes of the beads on the video frame were 43 µm in diameter under the magnification of $10 \times$. The initial locations and directions of movement of the synthetic images of the beads were randomly distributed over an area of $5.5 \times 4.0\;{\text{mm}}^{2}$ or $640 \times 480$ pixels, equivalently. Thus, the image of each synthetic bead occupied an area of $5 \times 5$ pixels. The speeds of the beads were set to follow a *Gaussian* speed distribution [36] with an average speed of $v_{\text{ave}}$ and a standard deviation of $\delta_{\text{set}}$ (µm/s). In this study, $v_{{{\text{ave}}\_{\text{set}}}}$ was fixed at 60 µm/s, close to the average curvilinear velocity of real sperms. Thus, $N_{\text{set}}$ and $\delta_{\text{set}}$ were the two parameters to be set for each of the 523 synthetic bead samples.

### Videos of synthetic bead samples

The movements of the moving beads in each synthetic bead sample were transformed into a video with a resolution of $640 \times 480$ pixels for 2 s at 60 Hz by using the LabVIEW 2014 and Vision Assistant 2014 programs. Afterward, the videos of the 523 synthetic bead samples were used to verify the validity of the proposed DFT method.

## Data Availability

Not applicable.

## Abbreviations

CASA: computer-assisted sperm analysis
CCD: charge-coupled device
CV: coefficient of variation
DFT: differential footprint trajectories
PCC: Pearson correlation coefficient
TMSC: total motile sperm count
TSC: total sperm count
WHO: World Health Organization
VCL: curvilinear velocity
